# Advancing the accuracy of clathrin protein prediction through multi-source protein language models

**DOI:** 10.1038/s41598-025-08510-4

**Published:** 2025-07-08

**Authors:** Watshara Shoombuatong, Nalini Schaduangrat, Pakpoom Mookdarsanit, Jaru Nikom, Lawankorn Mookdarsanit

**Affiliations:** 1 Center for Research Innovation and Biomedical Informatics, Faculty of Medical Technology, Mahidol University, Bangkok, 10700 Thailand; 2 Computer Science and Artificial Intelligence, Faculty of Science, Chandrakasem Rajabhat University, Bangkok, 10900 Thailand; 3 Research Methodology and Data Analytics Program, Faculty of Science and Technology, Prince of Songkla University, Pattani, 94000 Thailand; 4 Business Information System, Faculty of Management Science, Chandrakasem Rajabhat University, Bangkok, 10900 Thailand

**Keywords:** Clathrin, Sequence analysis, Bioinformatics, Protein language model, Machine learning, Feature selection, Deep learning

## Abstract

Clathrin is a key cytoplasmic protein that serves as the predominant structural element in the formation of coated vesicles. Specifically, clarithin enables the scission of newly formed vesicles from the plasma membrane’s cytoplasmic face. Efficient and accurate identification of clathrins is essential for understanding human diseases and aiding drug target development. Recent advancements in computational methods for identifying clathrins using sequence data have greatly improved large-scale clathrin screening. Here, we propose a high-accuracy computational approach, termed PLM-CLA, to achieve more accurate identification of clathrins. In PLM-CLA, we leveraged multi-source pre-trained protein language models (PLMs), which were trained on large-scale protein sequences from multiple database sources, including ProtT5-BFD, ProtT5-UR50, ProstT5, and ESM-2. These models were used to encode complementary feature embeddings, capturing diverse and valuable information. To the best of our knowledge, PLM-CLA is the first attempt designed using various PLM-based embeddings to identify clathrins. To enhance prediction performance, we utilized a feature selection method to optimize these fused feature embeddings. Finally, we employed a long short-term memory (LSTM) neural network model coupled with the optimal feature subset to identify clathrins. Benchmarking experiments, including independent tests, showed that PLM-CLA significantly outperformed state-of-the-art methods, achieving an accuracy of 0.961, MCC of 0.917, and AUC of 0.997. Furthermore, PLM-CLA secured outstanding performance in terms of MCC, with values of 0.971 and 0.904 on two existing independent test datasets. We anticipate that the proposed PLM-CLA model will serve as a promising tool for large-scale identification of clathrins in resource-limited settings.

**Supplementary Information:**

The online version contains supplementary material available at 10.1038/s41598-025-08510-4.

## Introduction

Clathrin, which is crucial for the cleavage of the membrane to release invaginated vesicles from the plasma membrane, manifests as a distinctive triskelion complex characterized by the association of three heavy chains at their C-terminal domains with corresponding light chains. As the fundamental constituent of vesicular coating assemblies, clathrin orchestrates crucial membrane trafficking events within the cytoplasmic milieu^1–3^. These clathrin-encapsulated vesicles execute precise cargo segregation across multiple cellular compartments, including the plasma membrane trans-Golgi network, and endosomal structures, facilitating diverse trafficking pathways. Upon cytoplasmic internalization, the clathrin lattice undergoes rapid disassembly, enabling protein recycling as vesicles proceed to various subcellular locations. This mechanistic pathway encompasses the selective internalization of diverse extracellular constituents, such as membrane receptors, ion channels, and proteins, all of which rely on clathrin-dependent endocytosis. Additionally, clathrin acts as the primary scaffolding element in the cellular uptake of DNA-chitosan nanoparticles and participates in cholesterol-enriched endocytosis processes, notably the caveolae-mediated pathway^4,5^. Extensive studies have shown that clathrin dysfunction significantly correlates with various pathological conditions, including Alzheimer’s disease, other neurodegenerative disorders, and malignant transformations like cancer^6–8^.

The critical role of clathrin proteins in human diseases has captivated numerous researchers, prompting extensive studies over the past decade. Various biological techniques have been implemented to identify clathrin proteins, such as using partial amino acid sequences^9^, employing the Tom1–Tollip complex^10^, or agarose gel electrophoresis^10^. In ground-breaking research, James et al.^11^ identified the clathrin-binding domain by proteolytically dividing AP-2 into light and heavy mero-AP components (LM-AP and HM-AP). Additionally, to further investigate clathrin-related proteins, scientists have discovered new clathrins, including assembly protein AP180^11^, γ2-adaptin^12^, TACC3/ch‐TOG/clathrin complex^13^, and myelin basic protein^14^.

Since experimental methods are expensive and labor-intensive, developing data-driven approaches, particularly in machine learning (ML) and deep learning (DL), is desirable for identifying clathrins in a cost-effective manner^15–21^. Generally, protein sequences are encoded into fixed-length feature vectors, which are then used to train and optimize ML models. These feature encodings are beneficial and directly impact model learning and performance. To date, several sequence-based feature encodings, well-regarded as hand-crafted feature encodings, have been employed for clathrin representation, such as position-specific scoring matrix (PSSM) profiles, encoding based from grouping weights (EBGW), dipeptide deviation from expected mean (DDE), and bigram PSSM (BiPSSM). For instance, Khanh Le et al. employed PSSM to represent clathrins and input the feature representation into a convolutional neural network (CNN) to build the first computational model, named deep-clathrin^22^. Overall, most existing methods, including deep-clathrin^22^, DeepCLA^23^, and CL-Pred^24^, were developed based on handcrafted features. However, these features may not provide sufficient information about the proteins. Moreover, the overall predictive performance of current methods is still not satisfactory for real-world prediction applications. To address this issue, a recent and effective approach inspired by natural language processing (NLP) is to utilize pre-trained protein language models (PLMs). Since PLMs are trained on large-scale sequence databases, this approach generates vital feature representations (feature embeddings) of protein sequences that are useful for downstream tasks, such as biological classification problems. To date, PLMs have demonstrated competitive and outstanding results compared to handcrafted feature encodings^25–29^.

In this study, we present PLM-CLA, a novel computational approach designed to accurately identify clathrins by leveraging multi-source PLMs from various database sources. In PLM-CLA, we constructed high-quality, non-redundant training and independent test datasets, referred to as CLA-TRN0.6 and CLA-IND0.6, respectively. Using these datasets, we employed four recent PLMs—ProtT5-BFD, ProtT5-UR50, ProstT5, and ESM-2—each pre-trained on different database sources to generate complementary feature embeddings. We then applied several popular feature section methods to determine the optimal feature subset. Finally, a long short-term memory (LSTM) neural network model was built using this optimal feature subset to develop the final PLM-CLA model. The major contributions of PLM-CLA compared to existing methods are as follows:

1. PLM-CLA is the first computational approach to utilize a combination of various PLM-based embeddings for clathrins identification;
2. The use of multi-source PLMs allows for automatic exploration of diverse and valuable information embedded within clathrins;
3. Extensive benchmarking experiments demonstrate that PLM-CLA consistently delivers stable performance across various benchmarked independent test datasets. Specifically, comparative experimental results indicate that PLM-CLA significantly outperforms existing methods on the CLA-IND0.6 dataset, achieving an accuracy (ACC) of 0.961, Matthew’s correlation coefficient (MCC) of 0.917, and an area under the receiver-operating curve (AUC) of 0.997. Furthermore, PLM-CLA exhibited impressive performance with MCC values of 0.971 and 0.904 on the independent tests conducted on CLA-IND1.0 and CLA-IND0.7, respectively.

## Materials and methods

### Dataset construction

Khanh Le et al.^22^ constructed the first benchmark dataset (referred to as Le2019) from the NCBI database^30^. In the Le2019 dataset, the training dataset (CLA-TRN1.0) included 1288 clathrins and 1133 non-clathrins, while the independent test dataset (CLA-IND1.0) contained 258 clathrins and 227 non-clathrins (Table 1). In 2020, Zhang et al. established another benchmark datasets (referred to as Zhang2020) to address sequence redundancy issues present in the Le2019 dataset. The Zhang2020 dataset construction involved three main steps: (i) collecting the original benchmark dataset with 1546 clathrins and 1360 non-clathrins from deep-clathrin^22^; (ii) removing sequence redundancy using a BLAST threshold of 0.7; (iii) excluding sequences with fewer than 220 residues; and (iv) oversampling the negative samples. The final Zhang2020 dataset consisted of 1347 clathrins and 1347 non-clathrins. Among these, 1212 clathrins, and 1212 non-clathrins were employed to create the training dataset (CLA-TRN0.7), while the remaining sequences formed the independent test dataset (CLA-IND0.7). Inspired by Zhang’s dataset, we further improved the benchmark dataset quality using a CD-HIT threshold of 0.6. This process resulted in 550 clathrins and 712 non-clathrins, named Shoombuatong2024. From this set, 1212 clathrins and 1212 non-clathrins were employed to construct the training dataset (CLA-TRN0.6), and the remaining sequences were used for the independent test dataset (CLA-IND0.6). The details of the datasets used for the development and performance evaluation of the existing methods are summarize in Table 1.

**Table 1:** A summary of the existing benchmark training and independent test datasets used for identifying clathrins.

Dataset	Training dataset		Independent test dataset		CD-HIT threshold	Dataset availability
	Positive	Negative	Positive	Negative		
Lee2019	1288	1133	258	227	1.0	Yes
Zhang2020	1212	1212	135	135	0.7	Yes
Khalid2024	542	550	278	221	0.25	No
Shoombuatong2024	480	602	69	110	0.6	Yes

### Protein language model

From the perspective of model development, feature extraction is crucial for creating effective and reliable models^27,28,31–33^. A wide range of feature extraction methods have been applied to biological classification problems, with handcrafted feature encodings being among the most popular. However, since these features are derived from a limited number of samples, they may fail to capture essential protein information^34–37^. To address this issue, one promising approach involves leveraging transformer-based language models (LMs) in NLP to transfer knowledge from vast unlabelled datasets to smaller, labelled datasets^37–41^. Inspired by these LMs, PLMs, trained on billions of protein sequences have been developed to generate feature representations (feature embeddings) that incorporate sequential, contextual, and structural information. This highlights that PLM-based feature embeddings process critical and diverse insights into protein structures and functions. In our study, we employed four PLMs (i.e., ProtT5-BFD, ProtT5-UR50, ProstT5, and ESM-2) to encode clathrins into feature embeddings. ProtT5-BFD and ProtT5-UR50 were developed by Rostlab based on the ProTrans framework^36^. Both these PLMs utilized the text-to-text transfer transformer (T5)^42^ architecture, employing teacher-forcing and span-generation methods. To be specific, ProtT5-UR50 was trained on Uniref50^43^, which consists of 45 million protein sequences, while ProtT5-BFD was trained on BFD^44^, a database containing 2.1 billion protein sequences. Unlike ProtT5-UR50 and ProtT5-BFD, ProstT5 applied span denoising and actual translation methods, using over 17 million sequences from the AlphaFold protein structure database^45^. For ESM-2, we used the esm2_t33_650M_UR50D model^46^, which is based on the BERT algorithm and was trained on Uniref50. Recently, these four PLMs have been successfully applied for downstream protein sequence tasks^27,29,38,47,48^. After feeding the input protein sequences into ProtT5-BFD, ProtT5-UR50, ProstT5, and ESM-2, we obtained feature embedding matrices with scales of $L\times 1024$, $L\times 1024$, $L\times 1024$, and $L\times 1280$, respectively. This demonstrates that these feature embeddings contain comprehensive and essential information about clathrins^35,38,47,49–51^.

### Feature selection method

In recent years, the curse of dimensionality is well-regarded as a big challenge in prediction and classification tasks. One effective way to mitigate this issue is through feature selection. The ultimate goal of feature selection is to exclude redundant features while identifying beneficial ones in order to enhance the predictive ability of the model^29,52–56^. Herein, we employed the elastic net (EN) method, introduced by Zou and Hastie, to select important features for training and optimizing our proposed model^57^. The EN method leverages *L*_1_ and *L*_2_ norms into a linear regression. To improve prediction performance, the EN method leverages both LASSO^58^ and Ridge regression^59^. Given a training dataset $\{({x}_{i}, {y}_{i}, i=1,\dots ,n)\}$, the linear regression is defined as:

1 $${\varvec{Y}}={\varvec{X}}\upbeta +\upvarepsilon$$

where ${\varvec{X}}=[{x}_{1},{x}_{2},\dots ,{x}_{n}]\in {\mathcal{R}}^{n\times p}$ and ${\varvec{Y}}=[{y}_{1},{y}_{2},\dots ,{y}_{n}]\in {\mathcal{R}}^{n}$. ${\varvec{X}}$ and ${\varvec{Y}}$ represent the data with *p* features and the response variable, respectively. Meanwhile, $\upbeta$ and $\upvarepsilon$ denote the vectors of coefficients and errors, respectively. Thus, estimating $\upbeta$ based on the EN method can be formulated as follows:

2 $$\underset{\upbeta }{\widehat{\upbeta }=\mathit{argmin}}(\frac{1}{n}{\Vert {\varvec{Y}}-{\varvec{X}}\upbeta \Vert }_{2}^{2}+\uplambda (1-{\upalpha }){\Vert \upbeta \Vert }^{2}+\uplambda {{\upalpha }\Vert \upbeta \Vert }_{1})$$

Here, the tuning parameter $\uplambda (\ge 0)$ controls shrinkage, while α is the regularization parameter that balances the contributions of LASSO and Ridge regression. As a result, the features with $\upbeta >0$ are considered important. In this study, the EN method was implemented using scikit-learn v1.5.2.

### Overall framework of PLM-CLA

**Fig. 1:**
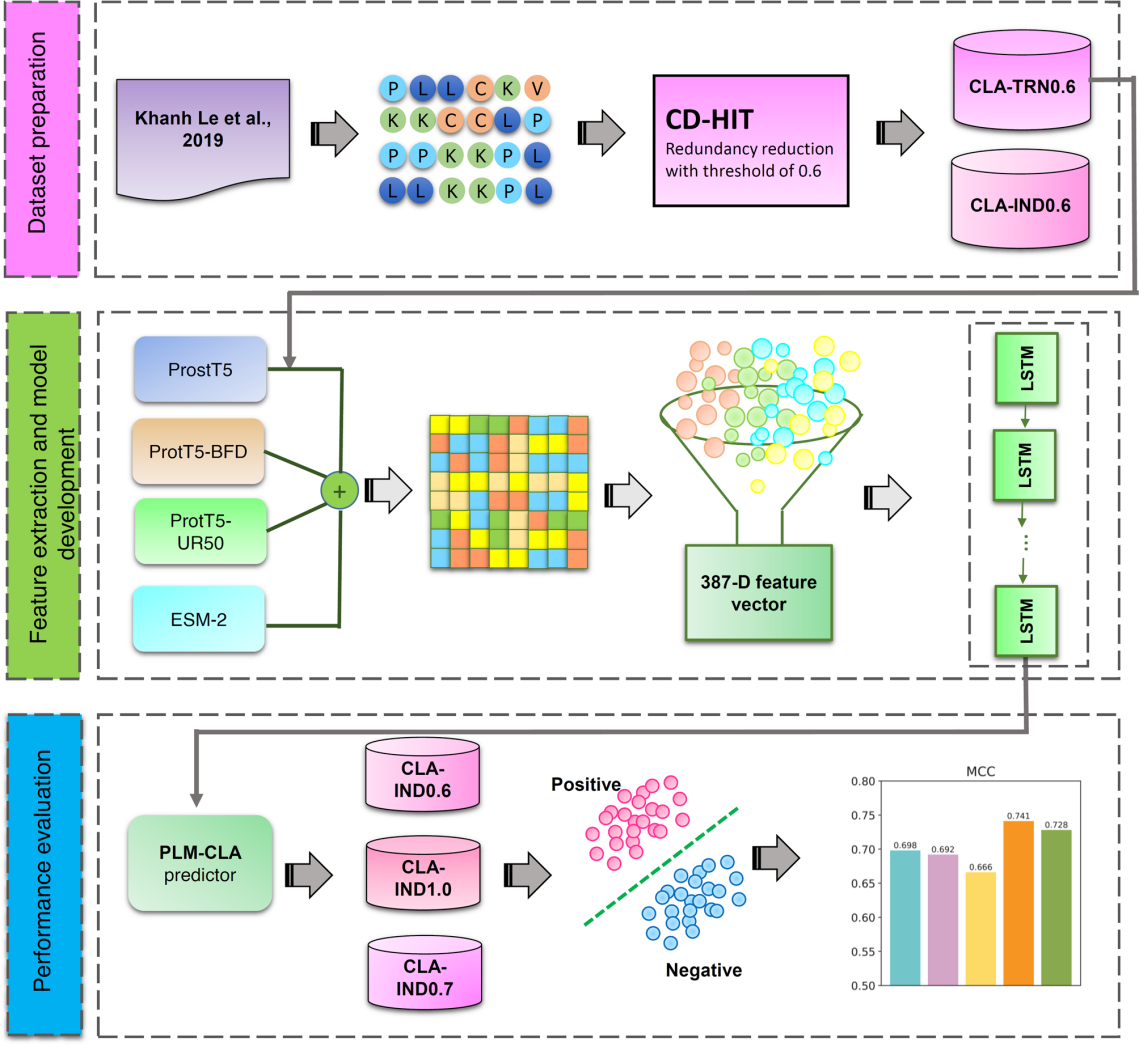
An overview of PLM-CLA framework for identifying clathrins. It involves the following steps: (i) dataset preparation, (ii) feature extraction, (iii) model development, and (iv) model evaluation.

As illustrated in Fig. 1 and Supplementary Figure S1, PLM-CLA is a DL-based approach for the identification of clathrins, where the input is a query protein sequence and the output is a probability score indicating the likelihood of being clathrin. The development of PLM-CLA consists of two main steps: (i) feature embedding extraction using multi-source PLM; and (ii) constructing an LSTM-based model for clathrin identification. In the first step, we encoded the protein sequences using ProtT5-BFD, ProtT5-UR50, ProstT5, and ESM-2 resulting in feature embeddings with dimensions of $L\times 1024$, $L\times 1024$, $L\times 1024$, and $L\times 1280$, respectively. To obtain more comprehensive information, we fused the feature embeddings from all four PLMs (referred to as Fusion). The Fusion was then fed into an LSTM method to generate the probability score for clathrin identification. LSTM, an advanced recurrent neural network (RNN)^60^, can learn and encode features from sequential input data. Although traditional RNNs are effective at learning from time series data, they have limitations in capturing long-term temporal correlations due to the vanishing gradient problem. LSTM addresses this issue by incorporating mechanisms such as the input gate ($i$), output gate ($o$), forget gate ($f$) and the cell candidate ($c$)^61,62^. Additionally, we applied various feature selection methods to the Fusion, including LASSO^58,63^, EN^57^, minimum redundancy maximum relevance (mRMR)^55,56^, principal component analysis (PCA)^48^, and max-relevance-max-distance (MRMD)^52^, to determine different feature subsets. Each feature subset was evaluated using LSTM in terms of cross-validation and independent test performance. Here, we selected the best-performing feature subset based on cross-validation MCC, for the development of our proposed model^31–33,64,65^. After obtaining the optimal feature subset, the ReLU function with a dropout rate of 0.5 was employed in the three output layers to generate prediction outputs (Supplementary Figure S1). Specifically, the softmax function was utilized as the activation function in the output layer to produce binary prediction outputs (i.e., clathrins and non-clathrins). The design and training of LSTM models were conducted using Tensorflow and Keras^66^ in the Python environment (Supplementary information).

### Performance evaluation

Herein, two standard evaluation strategies, namely ten-fold cross-validation and independent tests along with previous studies^15,16^, were employed to assess the clathrin protein prediction performance of PLM-CLA model. To avoid the overfitting and confirm the generalization ability, we applied the ten-fold cross-validation approach, where the training dataset was divided into ten subsets with equal size. In each iteration, nine subsets were employed for training, while the remaining subset was treated as the validation set. In addition, we applied the early stopping criterion to monitor the validation loss and stop training if the performance over the validation set failed to improve for a certain number of epochs. This process was repeated until each subset was used as the validation set. Finally, we obtained the ten prediction results from their corresponding validation sets and the cross-validation test results were obtained by computing the average results based on the ten prediction results.

We also employed the following six performance measures: ACC, AUC, MCC, F1, sensitivity (SN), and specificity (SP), to assess the predictive ability of our proposed model^29,31–33,64,65,67,68^ with the ten-fold cross-validation strategy. The corresponding equations for these measures are provided below:

3 $$\text{SN}=\frac{\text{TP}}{\left(\text{TP}+\text{FN}\right)}$$

4 $$\text{SP}=\frac{\text{TN}}{\left(\text{TN}+\text{FP}\right)}$$

5 $$\text{ACC}=\frac{\text{TP}+\text{TN}}{\left(\text{TP}+\text{TN}+\text{FP}+\text{FN}\right)}$$

6 $$\text{MCC}=\frac{\text{TP}\times \text{TN}-\text{FP}\times \text{FN}}{\sqrt[]{(\text{TP}+\text{FP})(\text{TP}+\text{FN})(\text{TN}+\text{FP})(\text{TN}+\text{FN})}}$$

7 $$\text{F}1=2\times \frac{\text{TP}}{2\text{TP}+\text{FP}+\text{FN}}$$

where true positives (TP) denote instances where the model accurately identifies positive cases, while true negatives (TN) represent correct identifications of negative cases. Conversely, false positives (FP) indicate cases erroneously classified as positive, and false negatives (FN) represent positive cases incorrectly labeled as negative^19–21,69^.

## Results and discussions

### Performance evaluation of individual feature embeddings

**Fig. 2:**
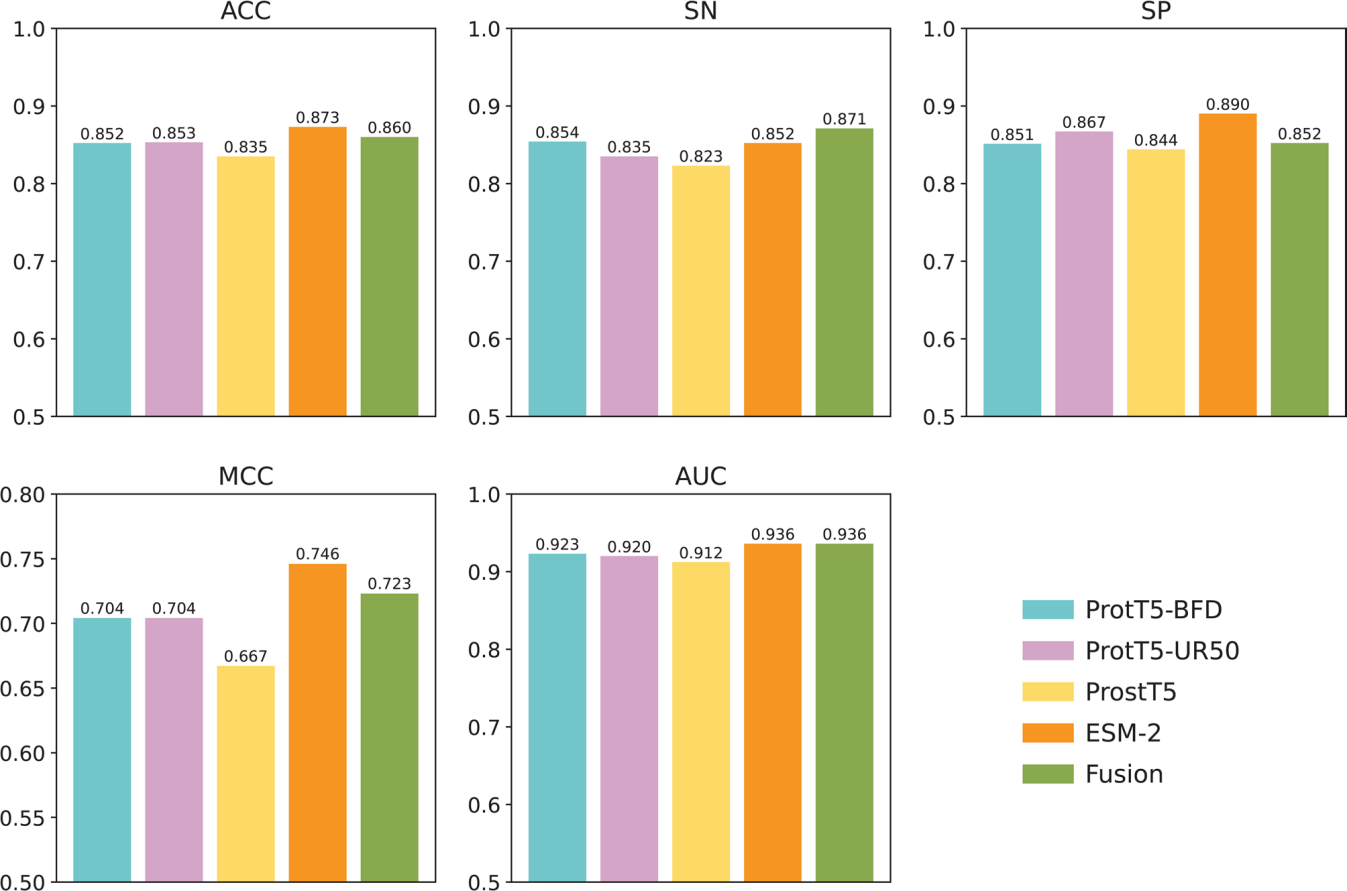
Performance comparison of six feature encoding schemes on the training dataset. Prediction results of six feature encoding schemes in terms of ACC, SN, SP, MCC, and AUC.

To assess the contribution of different feature embeddings in capturing crucial information, we trained variant LSTM models with four popular PLMs (i.e., ProstT5, ProtT5-BFD, ProtT5-UR50, and ESM-2). In addition, to obtain diverse information, we generate fused features (called Fusion) by combining all four PLM embeddings. The dimensions for ProstT5, ProtT5-BFD, and ProtT5-UR50 are 1024, while the dimensions for ESM-2 and Fusion are 1280 and 4352, respectively. Herein, the cross-validation procedure was applied to construct and evaluate each LSTM model, with performance evaluated in terms of ACC, F1, AUC, SN, SP, and MCC, as shown in Fig. 2 and Supplementary Table S1. Among the four individual feature embeddings, ESM-2 outperformed others across all performance measures. Specifically, ESM-2 achieved ACC, F1, AUC, SN, SP, and MCC values of 0.873, 0.857, 0.936, 0.852, 0.890, and 0.746, respectively. Meanwhile, Fusion demonstrated the second-best performance in terms of MCC. In the independent test, Fusion slightly outperformed ESM-2, in terms of ACC (0.860 versus 0.873) and MCC (0.847 versus 0.857). This finding indicates that combining multiple feature embeddings can provide crucial and complementary information, leading to more accurate identification of clathrin proteins.

### The effect of feature selection methods on the predictive performance

**Fig. 3:**
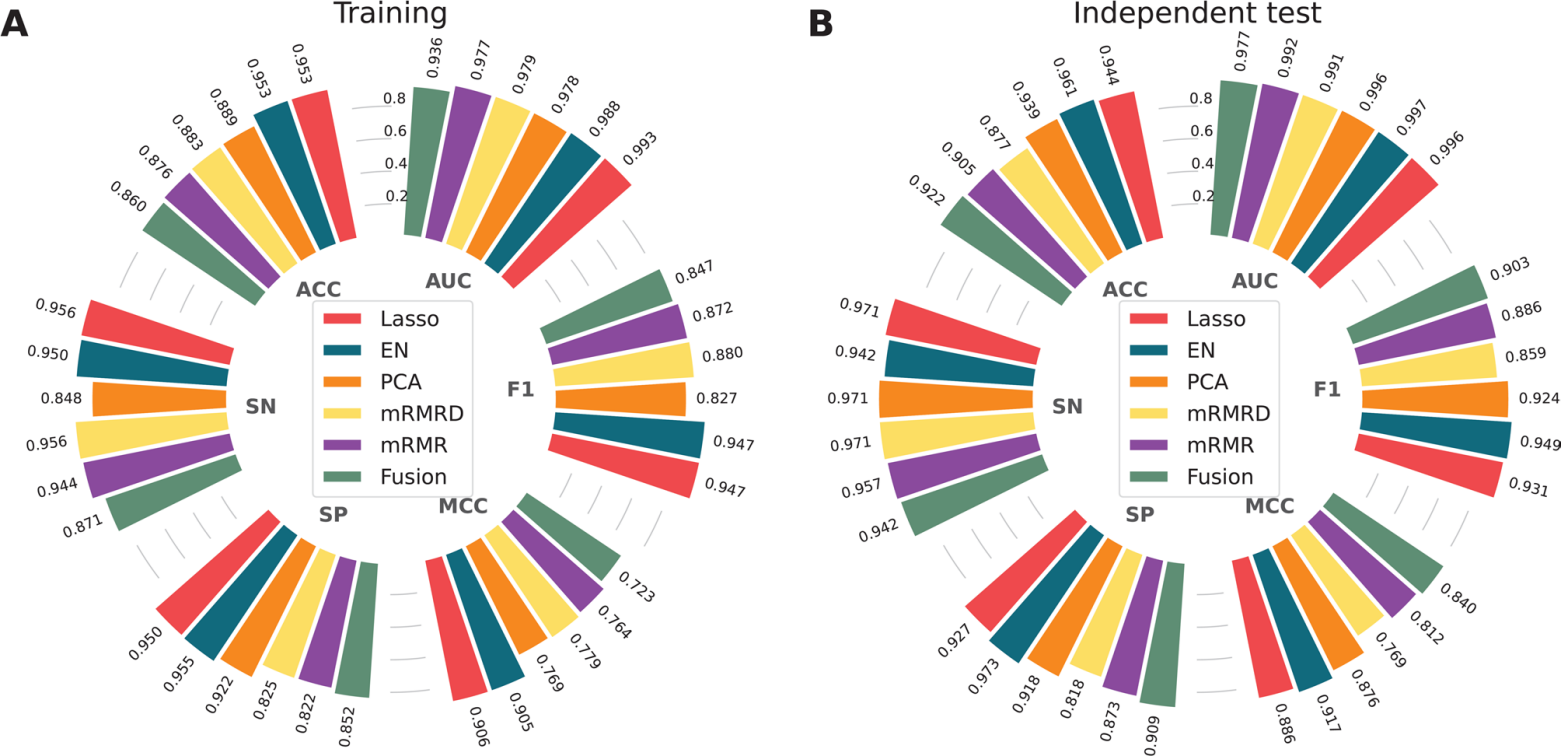
Performance comparison of different feature selection methods in terms of the training (**A**) and independent test (**B**) datasets.

**Fig. 4:**
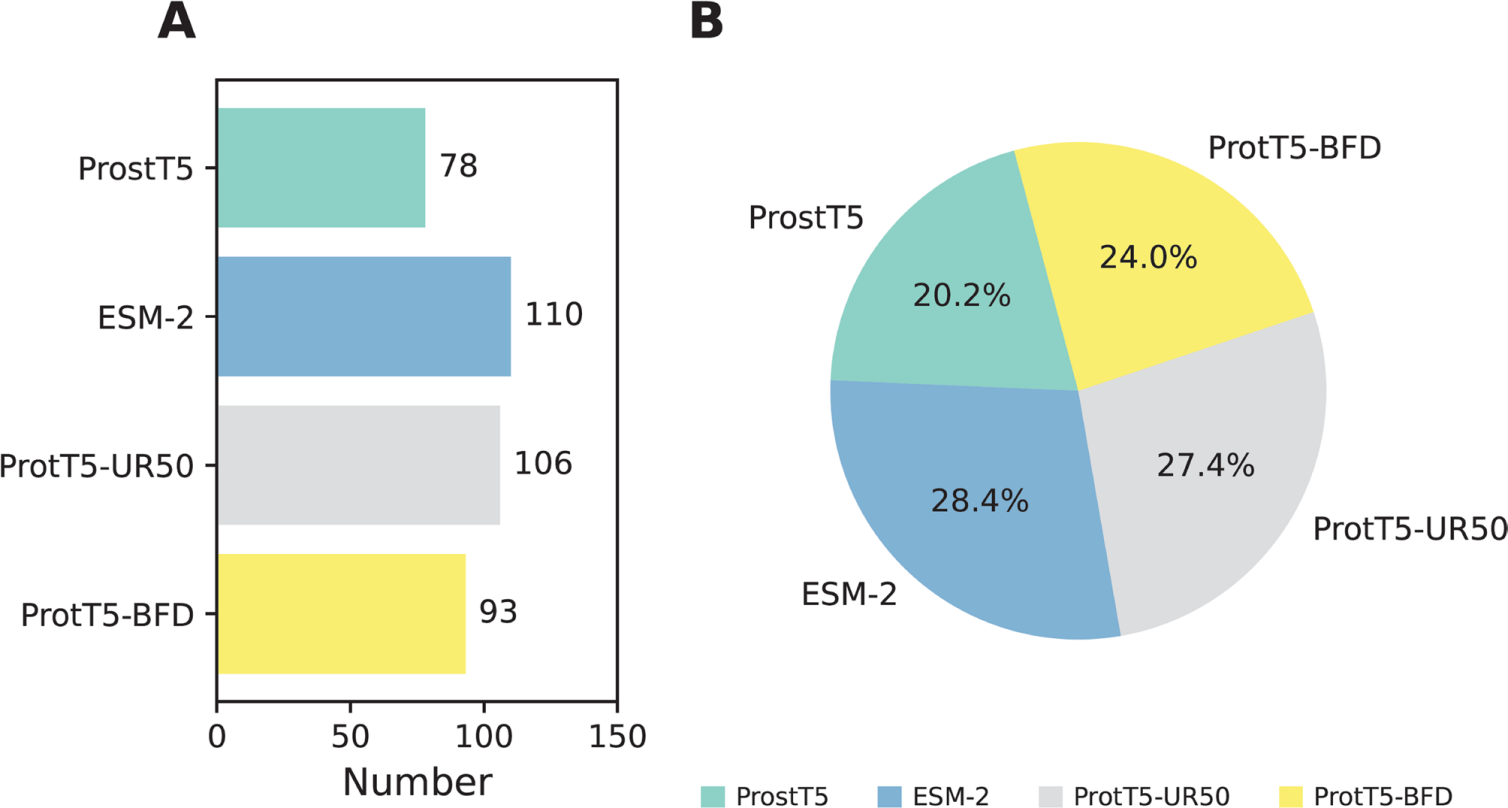
Analysis of the optimal feature set. The number (**A**) and proportion (**B**) of each type of selected feature embedding from the optimal feature set.

While the dimension of Fusion is 4,352, the size of the training dataset is only 1,082, which may hinder model learning and performance. To address this, feature selection is necessary. We applied five well-regarded feature selection methods for optimization, including LASSO, EN, mRMR, PCA, and MRMD. The optimal feature subset sizes derived from MRMD, mRMR, PCA, LASSO, and EN were 200, 200, 219, 229, and 387, respectively. The performance results, indicated by cross-validation and independent tests, are recorded in Fig. 3 and Supplementary Table S2. When comparing with Fusion, the cross-validation MCC values of the optimal feature subsets derived from MRMD, PCA, LASSO, and EN were superior. Notably, the LASSO-based and EN-based optimal feature subsets performed best, achieving MCC values in the range of 0.905–0.906 during cross-validation. These subsets showed ACC, MCC, and F1 values that were 9.24, 18.28–18.30, and 9.97–10.01% higher than those of Fusion, respectively. In the independent test, the EN-based optimal feature subset outperformed the LASSO subset across all five performance measures, with the sole exception of SN (0.942 versus 0.971). Overall, the EN-based feature subset (referred as EN_FS) significantly enhances the prediction performance of clathrin proteins, and thus, we applied it to design and optimize the LSTM model for the construction of PLM-CLA (Fig. 4).

### The necessity of combining multi-source PLMs

**Fig. 5:**
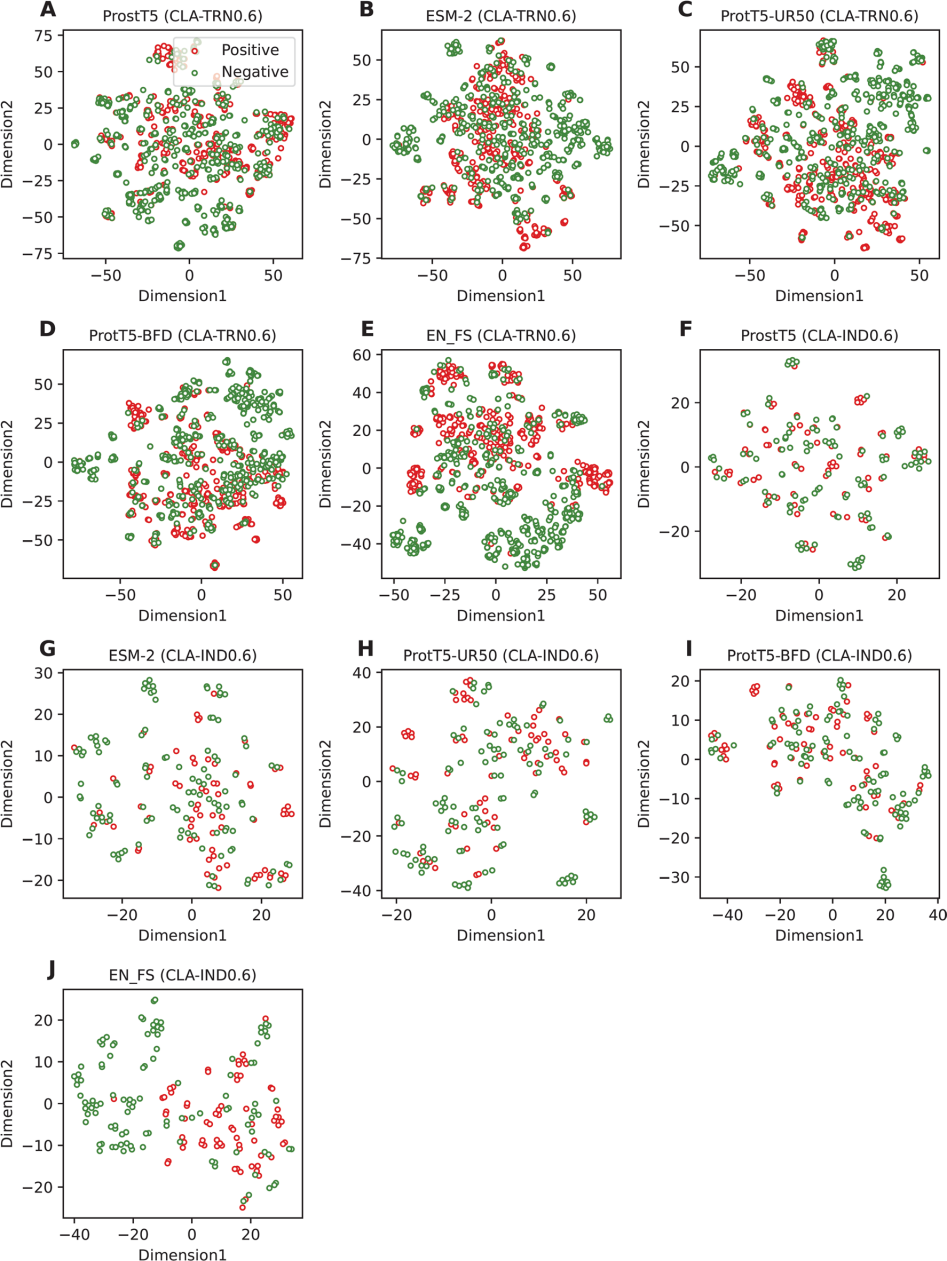
t-SNE visualization for different feature embeddings (i.e., ProstT5, ProtT5-BFD, ProtT5-UR50, and ESM-2) and our feature presentation (EN_FS) over the CLA-TRN0.6 (**A**–**E**) and CLA-IND0.6 (**F**–**J**) datasets.

To demonstrate the necessity of using multi-source PLMs, we compared the performance of our multi-view features (i.e., FS_EN) with single-feature embeddings (i.e., ProstT5, ProtT5-BFD, ProtT5-UR50, and ESM-2). The cross-validation and independent test results of FS_EN and the compared feature embeddings are summarized in Table 2. The experimental results clearly demonstrate that FS_EN achieved maximum effectiveness compared to the single-feature embeddings across all performance measures in both the cross-validation and independent tests. Specifically, the MCC values of FS_EN as judged by the cross-validation and independent tests, were 9.87 and 4.83% higher than the best-performing feature embedding (i.e., ESM-2), respectively. In addition, we applied t-Distributed Stochastic Neighbor Embedding (t-SNE)^32,33,65,70^ to compare the feature spaces of FS_EN and single-feature embeddings. This technique projects the original high-dimensional space into a two-dimensional space. We analyzed the feature spaces of ProstT5, ProtT5-BFD, ProtT5-UR50 (Fig. 5A–D,F–I), and FS_EN (Fig. 5E,J) using both the CLA-TRN0.6 and CLA-IND0.6 datasets. As evident in Fig. 5E,J, the positive and negative samples are more clearly distinguishable when using FS_EN. Overall, these results consistently highlight the necessity of combining multi-source PLMs to enhance prediction performance and feature representation, thereby achieving optimal clathrin identification performance.

**Table 2:** Performance of our proposed multi-view feature and single-feature embeddings over the cross-validation and independent tests.

Evaluation strategy	Embedding	ACC	SN	SP	MCC	AUC	F1
Cross-validation	ProtT5-UR	0.852	0.854	0.851	0.704	0.837	0.923
	ProtT5-BFD	0.853	0.835	0.867	0.704	0.834	0.920
	ProstT5	0.835	0.823	0.844	0.667	0.815	0.912
	ESM-2	0.873	0.852	0.890	0.746	0.857	0.936
	EN_FS	0.953	0.950	0.955	0.905	0.947	0.988
Independent test	ProtT5-UR	0.905	0.957	0.873	0.812	0.886	0.980
	ProtT5-BFD	0.922	0.899	0.936	0.835	0.899	0.974
	ProstT5	0.899	0.913	0.891	0.793	0.875	0.968
	ESM-2	0.916	0.913	0.918	0.825	0.894	0.958
	EN_FS	0.961	0.942	0.973	0.917	0.949	0.997

### Ablation experiments

As shown in Fig. 4, FS_EN consists of 78, 110, 106, and 93 features generated by ProstT5, ESM-2, ProtT5-UR50, and ProtT5-BFD, respectively. This distribution indicates that ESM-2 and ProtT5-UR50 together account for more than 50% of the selected features, while ProstT5 and ProtT5-BFD contribute 20.2 and 24.0%, respectively. In this section, the effect of each feature embedding on the identification of clathrins was evaluated through ablation experiments. Specifically, different variations of PLM-CLA were developed and tested for their predictive performance. Taking ProstT5 as an example, the PLM-CLA variant developed using 78 ProstT5 features was referred to as PLM-CLA (ProstT5). Herein, two standard evaluation strategies were employed to assess the performance of four PLM-CLA variants, and their prediction results are summarized in Supplementary Table S3. Overall, the results indicate that PLM-CLA outperformed its variations in terms of ACC, AUC, MCC, SP, MCC, and F1 across both cross-validation and independent tests. These findings confirm that incorporating diverse types of PLMs significantly enhances the model’s ability to achieve excellent classification performance in identifying clathrins.

### Comparison of PLM-CLA with well-known machine learning and deep learning models

**Fig. 6:**
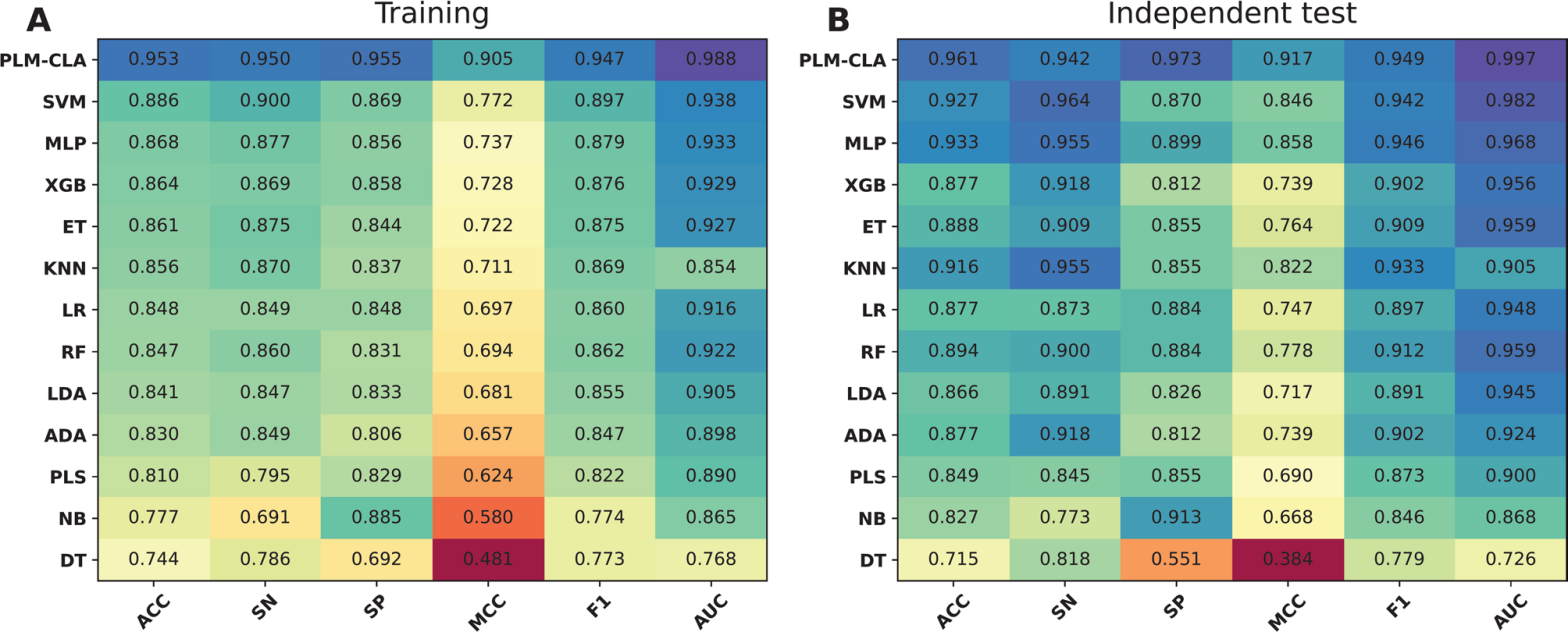
Heat-map of the prediction performance of PLM-CLA and conventional ML classifiers in terms of the training (**A**) and independent test (**B**) datasets.

**Fig. 7:**
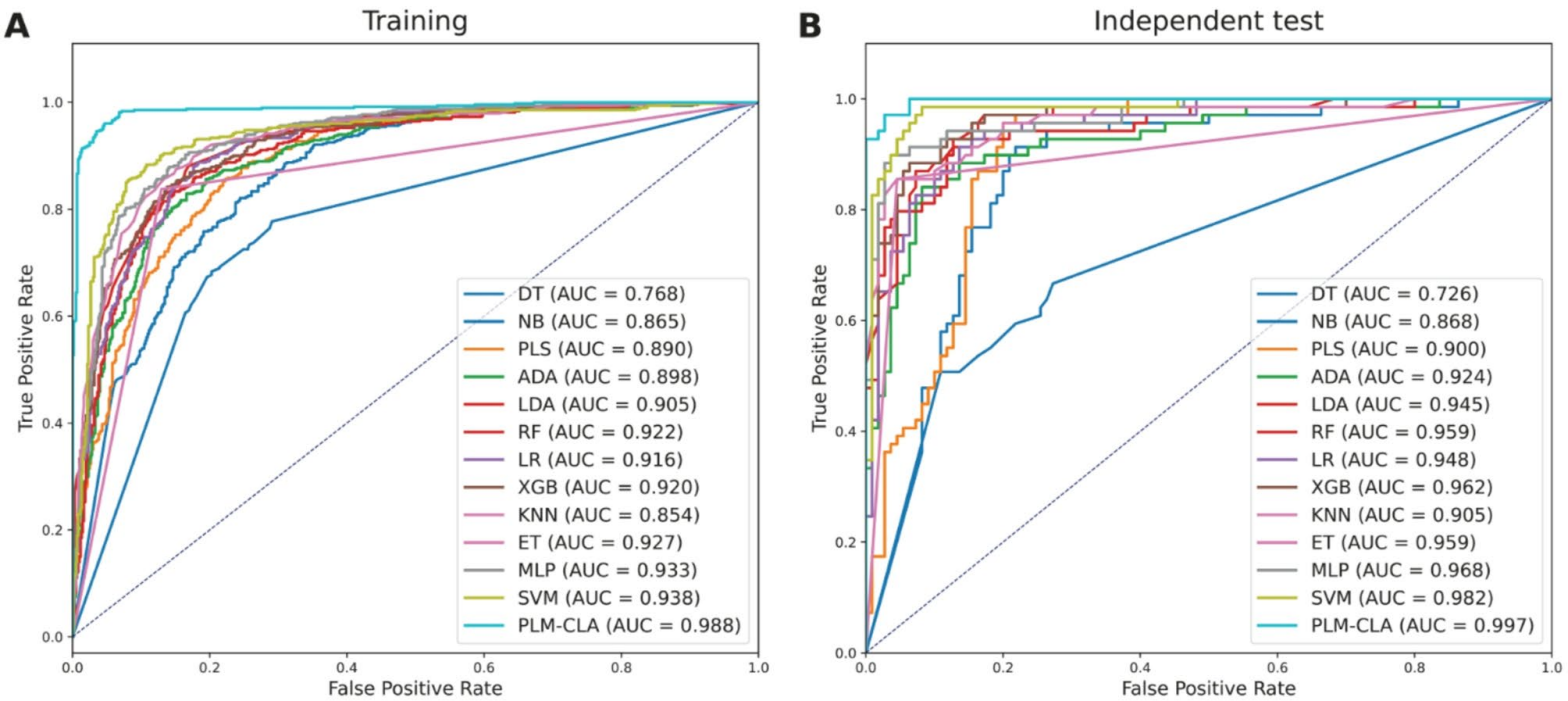
ROC curves illustrating the overall predictive performance of PLM-CLA and conventional ML classifiers in terms of the training (**A**) and independent test (**B**) datasets.

To evaluate the effectiveness and generalization ability of PLM-CLA, we conducted both cross-validation and independent tests using the CLA-TRN0.6 and CLA-IND0.6 datasets, respectively. We then compared its performance against several conventional ML models (i.e., DT, NB, PLS, ADA, LDA, RF, LR, KNN, ET, XGB, MLP, and SVM)^31,33,65^ and DL models (i.e., ResNet, CNN, DNN, and GRU)^63,71,72^. To ensure fair testing, these ML and DL models were constructed using the same feature subset and training dataset as PLM-CLA, with their optimal parameters tuned through a grid search strategy (Supplementary Tables S4-S5). The performance comparison results are presented in Figs. 6, 7, Tables 3, 4, and Supplementary Table S6. The top-five ML models with the highest cross-validation MCC values of 0.711, 0.722, 0.728, 0.737, 0.772 were KNN, ET, XGB, MLP, and SVM, respectively (Fig. 6A), while these models achieved corresponding MCC values of 0.822, 0.764, 0.739, 0.858, and 0.846, respectively, on the independent test (Fig. 6B). Based on both standard evaluation strategies, PLM-CLA exhibited the best performance compared to the top-five ML models across all performance measures, with the sole exception of SP in the independent test. To be specific, the MCC values of PLM-CLA were 0.905 and 0.917, representing improvements of 13.36–19.46 and 5.93–17.87% in the cross-validation and independent tests, respectively. When compared with four well-known DL methods, PLM-CLA exceeded the performance of ResNet, CNN, DNN, and GRU in terms of all performance measures on the independent test. As shown in Table 4, PLM-CLA’s MCC, ACC, F1, and AUC improvements ranged from 10.60–28.99, 5.03–13.97, 6.48–17.43, and 3.43–12.08%, respectively. Overall, PLM-CLA attained outstanding results and demonstrated consistent performance in identifying clathrins, showcasing its effectiveness and generalization ability.

**Table 3:** Performance comparison of PLM-CLA with conventional ML methods over the independent tests.

Method	ACC	SN	SP	MCC	F1	AUC
DT	0.715	0.818	0.551	0.384	0.779	0.726
NB	0.827	0.773	0.913	0.668	0.846	0.868
PLS	0.849	0.845	0.855	0.690	0.873	0.900
ADA	0.877	0.918	0.812	0.739	0.902	0.924
LDA	0.866	0.891	0.826	0.717	0.891	0.945
RF	0.894	0.900	0.884	0.778	0.912	0.959
LR	0.877	0.873	0.884	0.747	0.897	0.948
KNN	0.916	0.955	0.855	0.822	0.933	0.905
ET	0.888	0.909	0.855	0.764	0.909	0.959
XGB	0.877	0.918	0.812	0.739	0.902	0.956
MLP	0.933	0.955	0.899	0.858	0.946	0.968
SVM	0.927	0.964	0.870	0.846	0.942	0.982
PLM-CLA	0.961	0.942	0.973	0.917	0.949	0.997

**Table 4:** Performance comparison of PLM-CLA with conventional DL methods over the cross-validation and independent tests.

Evaluation strategy	Method	ACC	SN	SP	MCC	F1	AUC
Cross-validation	ResNet	0.767	0.731	0.796	0.529	0.735	0.840
	CNN	0.919	0.900	0.934	0.836	0.908	0.972
	DNN	0.925	0.921	0.929	0.849	0.916	0.975
	GRU	0.932	0.927	0.935	0.863	0.924	0.978
	PLM-CLA	0.953	0.950	0.955	0.905	0.947	0.988
Independent test	ResNet	0.821	0.797	0.836	0.627	0.775	0.876
	CNN	0.897	0.871	0.914	0.784	0.867	0.954
	DNN	0.911	0.884	0.927	0.811	0.884	0.963
	GRU	0.894	0.884	0.900	0.778	0.865	0.955
	PLM-CLA	0.961	0.942	0.973	0.917	0.949	0.997

### Comparison of PLM-CLA with existing methods

**Fig. 8:**
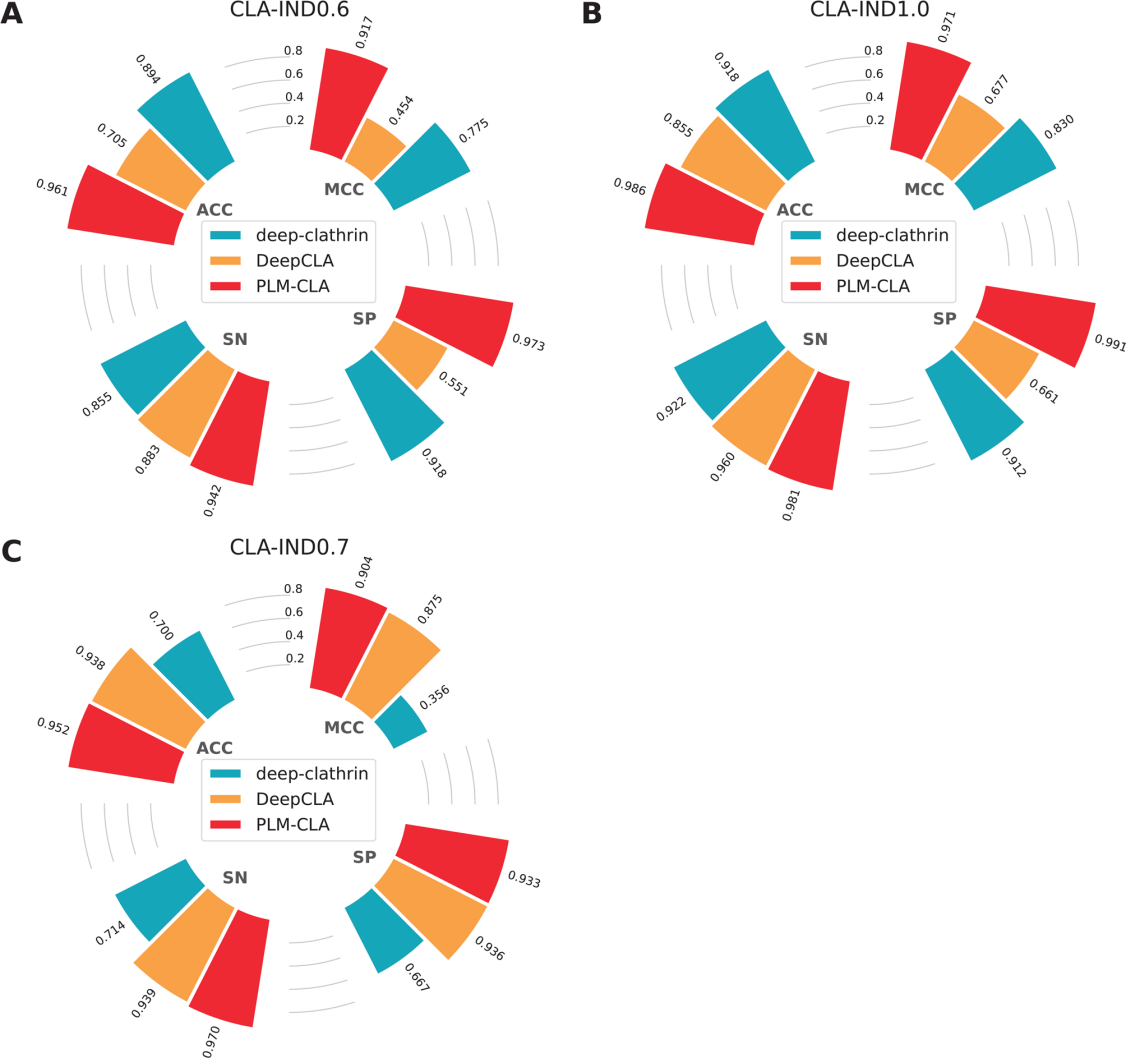
Performance comparison of PLM-CLA with the existing methods over the independent test on different independent test datasets. (**A**) CLA-IND0.6. (**B**) CLA-IND1.0. (**C**) CLA-IND0.7.

In this section, we compared PLM-CLA with existing methods, including deep-clathrin^22^, DeepCLA^23^, and CL-Pred^24^. Since the benchmark dataset used in this study differs from those used in developing the three existing methods, we reimplemented and evaluated the performance of these methods using the CLA-TRN0.6 and CLA-IND0.6 datasets. As CL-Pred did not provide source code for clathrin prediction, we only assessed the performance of deep-clathrin and DeepCLA in this study. Figure 8A and Table 5 summarize performance comparison results on the independent test using CLA-IND0.6. As shown, PLM-CLA outperformed both deep-clathrin and DeepCLA in terms of ACC, AUC, SN, SP, and MCC. Specifically, the ACC, SN, SP, and MCC values of PLM-CLA were 6.69, 8.70, 5.47, 14.23% higher, respectively, than those of the second-based model (i.e., deep-clathrin). Altogether, these results indicate that our proposed model demonstrates strong generalization ability and robustness.

**Table 5:** Performance comparison of DLPLM-CLA with the existing methods on various benchmark datasets over the independent test.

Dataset	Method	ACC	SN	SP	MCC	AUC
CLA-IND1.0	deep-clathrin	0.918	0.922	0.912	0.830	–
	DeepCLA	0.855	0.960	0.661	0.677	0.913
	PLM-CLA	0.986	0.981	0.991	0.971	0.995
CLA-IND7.0	deep-clathrin	0.700	0.714	0.667	0.356	0.692
	DeepCLA	0.938	0.939	0.936	0.875	0.937
	PLM-CLA	0.952	0.970	0.933	0.904	0.991
CLA-IND0.6	deep-clathrin	0.894	0.855	0.918	0.775	0.942
	DeepCLA	0.705	0.883	0.551	0.454	0.820
	PLM-CLA	0.961	0.942	0.973	0.917	0.997

### Performance evaluation of PLM-CLA on other benchmark independent test datasets

Here, we aim to develop a novel prediction model that can maintain stable performance across various independent test datasets. To evaluate the proposed PLM-CLA model’s effectiveness, we tested it on available benchmarks datasets, including CLA-IND1.0 and CLA-IND0.7 datasets (as detailed in Fig. 8 and Table 5). We also compared our model with existing methods that provided source codes for clathrin prediction, such as deep-clathrin^22^ and DeepCLA^23^. First, we evaluated the performance of PLM-CLA over the first independent test dataset (CLA-IND1.0), originally collected by Khanh Le et al.^22^. The performance metrics of deep-clathrin was directly obtained from the literature^22^. For DeepCLA, we implemented a hybrid DL-based model, integrating CNN and Bi-LSTM networks with their specific hyperparameters on the CLA-TRN1.0 dataset. Its independent test performance was then evaluated on the CLA-IND1.0 dataset. Figure 8B shows the performance of deep-clathrin, DeepCLA, and PLM-CLA on the independent test. As can be seen from Fig. 8B, PLM-CLA outperformed both deep-clathrin and DeepCLA in terms of ACC, SP, MCC, and AUC, with deep-clathrin achieving the second-best MCC value. To be specific, the ACC, SN, SP, and MCC values of PLM-CLA were 6.76, 5.86, 7.92, and 14.11% higher than the second-best model. When evaluating the performance on the CLA-IND0.7 dataset, prediction results for deep-clathrin and DeepCLA^23^ were directly obtained from the literature. As shown in Fig. 8C, both DeepCLA and PLM-CLA exhibited similar performance and surpassed deep-clathrin in terms of ACC, SN, SP, MCC, and AUC on the independent test. Although PLM-CLA’s SP value (0.933 versus 0.936) was slightly lower than DeepCLA’s on the CLA-IND0.7 dataset, PLM-CLA significantly outperformed DeepCLA in MCC on both the CLA-IND1.0 (0.971 versus 0.677) and on the CLA-IND0.6 (0.917 versus 0.454) datasets. Considering results across all three independent test datasets (Fig. 8), the proposed PLM-CLA model attained improved performance, indicating its stability and generalization capability.

## Conclusion

This study proposes PLM-CLA, the first PLM-based computational approach for accurately identifying clathrins. In PLM-CLA, we leveraged ProtT5-BFD, ProtT5-UR50, ProstT5, and ESM-2, which were trained on multi-source protein databases, to generate the multi-view information embedded in clathrins. Consequently, an optimized computational approach was constructed, and its prediction performance was evaluated through cross-validation and independent tests on multiple training and independent test datasets. Compared with two existing methods (i.e., deep-clathrin and DeepCLA) across three independent test datasets, PLM-CLA demonstrated stable performance, achieving MCC values of 0.917, 0.971, and 0.904 on the CLA-IND0.6, CLA-IND1.0, and CLA-IND0.7 datasets, respectively. These results undervalue the effectiveness and generalization ability of the proposed computational approach. The high performance of PLM-CLA can be attributed to two key factors: (i) leveraging the advantages of multi-source PLM models to generate comprehensive and essential information about clathrins; and (ii) conducting a thorough investigated of multiple feature subset to determine the optimal one, ensuring high performance in clathrin identification. We hope that the proposed PLM-CLA model can facilitate large-scale clathrin identification in resource-limited settings. Although PLM-CLA has attained significant performance improvements, there is still room for optimization in our future work. Firstly, to obtain a more comprehensive and high-quality dataset, we plan to collect additional clathrin sequences and integrate them into the existing benchmark dataset. Secondly, interpretable feature descriptors, such as amino acid and dipeptide propensities^73,74^, will be employed to enhance the interpretability of PLM-CLA. These descriptors will be extracted using a propensity score representation learning scheme and employed to ascertain critical physicochemical attributes of clathrins. Thirdly, we plan to integrate PLM-CLA with novel and effective ML frameworks, such as complex-valued polynomial model^75^, multi-step stacking strategy^64,76^, and voting transfer learning strategy^77^. Lastly, we will develop a web-accessible computational resource to facilitate the community-wide use of PLM-CLA.

## Electronic supplementary material

Below is the link to the electronic supplementary material.

Supplementary Material 1

## Data Availability

All datasets and source codes of PLM-CLA are available at https://github.com/lawankorn-m/Clathrin/tree/main
