# Supplementary material for: Advancing the accuracy of clathrin protein prediction through multi-source protein language models
Source: Sci Rep. 2025 Jul 8;15:24403. doi: 10.1038/s41598-025-08510-4 (PMC12238356; doi:10.1038/s41598-025-08510-4)
Supplement: Supplementary file 1 — Supplementary Material 1 [file 41598_2025_8510_MOESM1_ESM.docx]

## Supplementary information

The PLM-CLA has two main sub-networks. The first network encoded protein sequences into a fixed-length vector, while the last network generate the prediction output. In PLM-CLA, LSTM transformed the input embedding matrices into a fixed-length vector. Specifically, the 193-dimensional vectors (i.e., EN_FS) generated from the first LSTM layer were passed to the next LSTM layer, which outputed 96-dimensional vectors. The dimensions of the LSTM layers were reduced in the order of 193 and 96 across two layers. A dropout rate of 0.5 was applied in each LSTM layer to alleviate the overfitting issue. The outputs were flattened before passing through three dense layers. The dimensions of the dense layers were reduced in the order of 96, 48, and 1. The ReLU function with a dropout rate of 0.5 was employed in the three output layers to obtain the prediction outputs. Specifically, we utilized the softmax function as an activation function at the output layer to generate binary prediction outputs (i.e., clathrins and non-clathrins). The design and learning of LSTM models were conducted based on using Tensorflow and Keras of the python package.

## Supplementary Figure

**
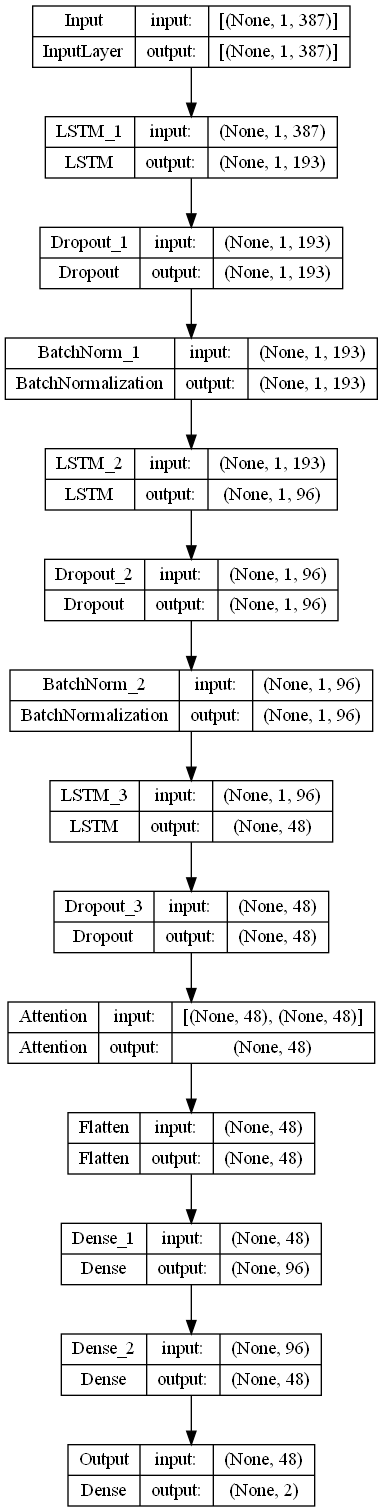
**

## Figure S1 Overall structure of PLM-CLA. The clathrin sequences were encoded into fixed-length vectors using ProtT5-BFD, ProtT5-UR50, ProstT5, and ESM-2. Then, the feature selection method was used to determine the optimal feature subset. Finally, the prediction outputs were obtained by using three fully connected layers surrounded by the purple line.

## Supplementary Table

## Table S1 Performance of different feature embeddings over the cross-validation test.

| **Embedding** | **ACC** | **SN** | **SP** | **MCC** | **F1** | **AUC** |
| --- | --- | --- | --- | --- | --- | --- |
| ProtT5-UR | 0.852 | 0.854 | 0.851 | 0.704 | 0.837 | 0.923 |
| ProtT5-BFD | 0.853 | 0.835 | 0.867 | 0.704 | 0.834 | 0.920 |
| ProstT5 | 0.835 | 0.823 | 0.844 | 0.667 | 0.815 | 0.912 |
| ESM-2 | 0.873 | 0.852 | 0.890 | 0.746 | 0.857 | 0.936 |
| Fusion | 0.860 | 0.871 | 0.852 | 0.723 | 0.847 | 0.936 |

## Table S2 Performance comparison of different feature selection methods over the cross-validation and independent tests.

| **Evaluation strategy** | **Method** | **ACC** | **SN** | **SP** | **MCC** | **F1** | **AUC** |
| --- | --- | --- | --- | --- | --- | --- | --- |
| Cross-validation | Original | 0.860 | 0.871 | 0.852 | 0.723 | 0.847 | 0.936 |
|  | mRMRD | 0.883 | 0.956 | 0.825 | 0.779 | 0.880 | 0.979 |
|  | mRMR | 0.876 | 0.944 | 0.822 | 0.764 | 0.872 | 0.977 |
|  | PCA | 0.889 | 0.848 | 0.922 | 0.769 | 0.827 | 0.978 |
|  | LASSO | 0.953 | 0.956 | 0.950 | 0.906 | 0.947 | 0.993 |
|  | EN | 0.953 | 0.950 | 0.955 | 0.905 | 0.947 | 0.988 |
| Independent test | Original | 0.922 | 0.942 | 0.909 | 0.840 | 0.903 | 0.977 |
|  | mRMRD | 0.877 | 0.971 | 0.818 | 0.769 | 0.859 | 0.991 |
|  | mRMR | 0.905 | 0.957 | 0.873 | 0.812 | 0.886 | 0.992 |
|  | PCA | 0.939 | 0.971 | 0.918 | 0.876 | 0.924 | 0.996 |
|  | LASSO | 0.944 | 0.971 | 0.927 | 0.886 | 0.931 | 0.996 |
|  | EN | 0.961 | 0.942 | 0.973 | 0.917 | 0.949 | 0.997 |

## Table S3 Performance comparison of different variations of PLM-CLA in the ablation experiment over the cross-validation and independent tests.

| **Evaluation strategy** | **Method** | **ACC** | **SN** | **SP** | **MCC** | **F1** | **AUC** |
| --- | --- | --- | --- | --- | --- | --- | --- |
| Cross-validation | PLM-CLA (ProtT5-UR50) | 0.866 | 0.971 | 0.782 | 0.757 | 0.868 | 0.980 |
|  | PLM-CLA (ProtT5-BFD) | 0.910 | 0.946 | 0.882 | 0.827 | 0.905 | 0.980 |
|  | PLM-CLA (ProstT5) | 0.769 | 0.667 | 0.850 | 0.524 | 0.605 | 0.943 |
|  | PLM-CLA (ESM-2) | 0.823 | 0.858 | 0.794 | 0.658 | 0.769 | 0.973 |
|  | PLM-CLA | 0.953 | 0.950 | 0.955 | 0.905 | 0.947 | 0.988 |
| Independent test | PLM-CLA (ProtT5-UR50) | 0.894 | 0.986 | 0.836 | 0.801 | 0.877 | 0.991 |
|  | PLM-CLA (ProtT5-BFD) | 0.916 | 0.928 | 0.909 | 0.827 | 0.895 | 0.988 |
|  | PLM-CLA (ProstT5) | 0.911 | 0.928 | 0.900 | 0.816 | 0.889 | 0.986 |
|  | PLM-CLA (ESM-2) | 0.883 | 0.971 | 0.827 | 0.778 | 0.865 | 0.984 |
|  | PLM-CLA | 0.961 | 0.942 | 0.973 | 0.917 | 0.949 | 0.997 |

## Table S4 Information of parameter settings for 12 ML methods used in this study.

| **Method** | **Parameter** | **Search space** |
| --- | --- | --- |
| ADA | n_estimators | [20, 50, 100, 200, 500] |
| DT | max_depth | 2–20 with an interval of 1. |
| ET | n_estimators | [20, 50, 100, 200, 500] |
| KNN | number of neighbours | 1–150 with an interval of 1 |
| LGBM | n_estimators | [20, 50, 100, 200, 500] |
| LR | C | np.logspace(-3, 3, num=100) |
| MLP | hidden_layer_sizes | [20, 50, 100, 200, 500] |
| NB | var_smoothing | np.logspace(0,-9, num=100) |
| PLS | #Components | 10–1000 with an interval of 10 |
| RF | n_estimators | [20, 50, 100, 200, 500] |
| SVM | Cost | [2^-4^–2^4^] in log_2_ steps |
| XGB | n_estimators | [20, 50, 100, 200, 500] |

Columns 2 and 3 represents the parameter name used in the Scikit-learn library and the range of parameter used to develop the model, respectively.

## Table S5 Information of hyper-parameter settings for five DL methods used in this study.

| **Method** | **Parameter** | **Search space** |
| --- | --- | --- |
| ResNet | Filters (Conv1D) | [64, 128, 256] |
|  | Batch Normalization | True |
|  | Residual Blocks | [64, 128, 256] |
|  | Kernel Size | [3] |
|  | Activation | ['relu', ‘softmax’] |
|  | Optimizer | ['adam'] |
|  | Kernel Regularizer | [l2(0.0001)] |
|  | Learning Rate | [0.001, 0.01, 0.1] |
|  | Dropout Rate | [0.2, 0.3, 0.4, 0.5] |
|  | Loss Function | ['binary_crossentropy'] |
| LSTM | Units | [16, 32, 64, 128] |
|  | Dropout Rate | [0.5] |
|  | Activation | ['relu', 'softmax'] |
|  | Optimizer | ['adam''] |
|  | Learning Rate | [0.001, 0.01, 0.1] |
|  | Loss Function | ['categorical_crossentropy'] |
| GRU | Units | [64, 128, 256] |
|  | Dropout Rate | [0.5] |
|  | Activation | ['relu', 'softmax'] |
|  | Optimizer | ['adam''] |
|  | Learning Rate | [0.001, 0.01, 0.1] |
|  | Loss Function | ['categorical_crossentropy'] |
| CNN | Filters (Conv1D) | [32, 32] |
|  | Kernel Size | [3] |
|  | Activation | ['relu', ‘softmax’] |
|  | Optimizer | ['adam'] |
|  | Learning Rate | [0.001, 0.01, 0.1] |
|  | Loss Function | ['categorical_crossentropy'] |
| DNN | Hidden_layers | [1, 2, 3, 4] |
|  | Units_per_layer | [16, 32, 64, 128, 256, 512] |
|  | Activation | ['relu', 'softmax'] |
|  | Learning Rate | [0.001, 0.01, 0.1] |
|  | Dropout_rate | [0.5] |
|  | Loss Function | ['categorical_crossentropy'] |

## Table S6 Performance comparison of PLM-CLA with conventional ML methods over the ten-fold cross-validation test

| **Method** | **ACC** | **SN** | **SP** | **MCC** | **F1** | **AUC** |
| --- | --- | --- | --- | --- | --- | --- |
| DT | 0.744 | 0.786 | 0.692 | 0.481 | 0.773 | 0.768 |
| NB | 0.777 | 0.691 | 0.885 | 0.580 | 0.774 | 0.865 |
| PLS | 0.810 | 0.795 | 0.829 | 0.624 | 0.822 | 0.890 |
| ADA | 0.830 | 0.849 | 0.806 | 0.657 | 0.847 | 0.898 |
| LDA | 0.841 | 0.847 | 0.833 | 0.681 | 0.855 | 0.905 |
| RF | 0.847 | 0.860 | 0.831 | 0.694 | 0.862 | 0.922 |
| LR | 0.848 | 0.849 | 0.848 | 0.697 | 0.860 | 0.916 |
| KNN | 0.856 | 0.870 | 0.837 | 0.711 | 0.869 | 0.854 |
| ET | 0.861 | 0.875 | 0.844 | 0.722 | 0.875 | 0.927 |
| XGB | 0.864 | 0.869 | 0.858 | 0.728 | 0.876 | 0.929 |
| MLP | 0.868 | 0.877 | 0.856 | 0.737 | 0.879 | 0.933 |
| SVM | 0.886 | 0.900 | 0.869 | 0.772 | 0.897 | 0.938 |
| PLM-CLA | 0.953 | 0.950 | 0.955 | 0.905 | 0.947 | 0.988 |
